# The potential role of the thymus in immunotherapies for acute myeloid leukemia

**DOI:** 10.3389/fimmu.2023.1102517

**Published:** 2023-02-06

**Authors:** Christopher Hino, Yi Xu, Jeffrey Xiao, David J. Baylink, Mark E. Reeves, Huynh Cao

**Affiliations:** ^1^ Department of Internal Medicine, Loma Linda University, Loma Linda, CA, United States; ^2^ Division of Hematology and Oncology, Department of Medicine, Loma Linda University, Loma Linda, CA, United States; ^3^ Division of Regenerative Medicine, Department of Medicine, Loma Linda University, Loma Linda, CA, United States; ^4^ Loma Linda University Cancer Center, Loma Linda, CA, United States

**Keywords:** AML-acute myeloid leukemia, thymus, immunotherapy, T lymphocytes, aging, immunosenescence, hematopoietic (stem) cell transplant (HCST), tumor microenvironment

## Abstract

Understanding the factors which shape T-lymphocyte immunity is critical for the development and application of future immunotherapeutic strategies in treating hematological malignancies. The thymus, a specialized central lymphoid organ, plays important roles in generating a diverse T lymphocyte repertoire during the infantile and juvenile stages of humans. However, age-associated thymic involution and diseases or treatment associated injury result in a decline in its continuous role in the maintenance of T cell-mediated anti-tumor/virus immunity. Acute myeloid leukemia (AML) is an aggressive hematologic malignancy that mainly affects older adults, and the disease’s progression is known to consist of an impaired immune surveillance including a reduction in naïve T cell output, a restriction in T cell receptor repertoire, and an increase in frequencies of regulatory T cells. As one of the most successful immunotherapies thus far developed for malignancy, T-cell-based adoptive cell therapies could be essential for the development of a durable effective treatment to eliminate residue leukemic cells (blasts) and prevent AML relapse. Thus, a detailed cellular and molecular landscape of how the adult thymus functions within the context of the AML microenvironment will provide new insights into both the immune-related pathogenesis and the regeneration of a functional immune system against leukemia in AML patients. Herein, we review the available evidence supporting the potential correlation between thymic dysfunction and T-lymphocyte impairment with the ontogeny of AML (II-VI). We then discuss how the thymus could impact current and future therapeutic approaches in AML (VII). Finally, we review various strategies to rejuvenate thymic function to improve the precision and efficacy of cancer immunotherapy (VIII).

## 1 Introduction

Cancer immunotherapy has revolutionized the treatment landscape for different tumors and has demonstrated the feasibility of leveraging the host’s immune system to control and eliminate malignant cells (1). In hematological cancers, the discovery of the graft-vs-leukemia (GVL) effect after allogeneic hematopoietic stem cell transplantation (allo-HSCT) demonstrated that leukemia is susceptible to immune-mediated eradication (2). More recently in the last decade, the development of immune checkpoint inhibitors (ICIs) and genetically engineered chimeric antigen receptor (CAR)-T cell therapy has resulted in promising therapeutic responses in certain hematological malignancies (3–7).

T lymphocytes are major contributors to the success of these immunotherapies; however, optimal responses rely on the persistent availability of a diverse and functional T lymphocyte repertoire capable of recognizing tumor neo-antigens and eliciting cytotoxicity (8). The thymus constitutes of a lymphoid organ dedicated to generating new functional T lymphocytes and ‘educating’ them to recognize a wide diversity of tumor and pathogenic antigens while simultaneously avoiding self-reactivity (9). However, this process is significantly impaired in the adult thymus as a result of age and/or environmental insults, such as infections or cytoreductive therapies, and it may have serious consequences on the clinical efficacy of immune-based therapies (10).

Despite the success of allo-HSCT to treat younger patients, the treatment of acute myeloid leukemia (AML) in older patients and those with relapsed or refractory disease remains challenging in part due to poor immune T-cell responses (11). An emerging body of evidence has shown that AML patients have numerically and functionally defective T cells and NK cells at the time of diagnosis (12–14). The variable efficacy of T cell-based immunotherapeutic strategies underscores the compelling need to decipher the factors which shape the T-cell landscape in AML.

To date, major efforts have been made to understand the immune landscape within the context of the bone marrow microenvironment in AML (15, 16). However, the role of the thymic microenvironment in propagating AML progression is largely underrecognized, but a potentially important aspect of AML biology. In this review, we 1) provide an overview of our current knowledge of T cell development in the thymus (II-VI), 2) link the potential association between T-lymphocyte impairment caused by thymus dysfunction and the ontogeny of AML (VII), and 3) hypothesize how thymic regeneration may improve future immunotherapeutic strategies for the treatment of AML (VIII).

## 2 The role of thymus in T cell development

**Figure 1:**
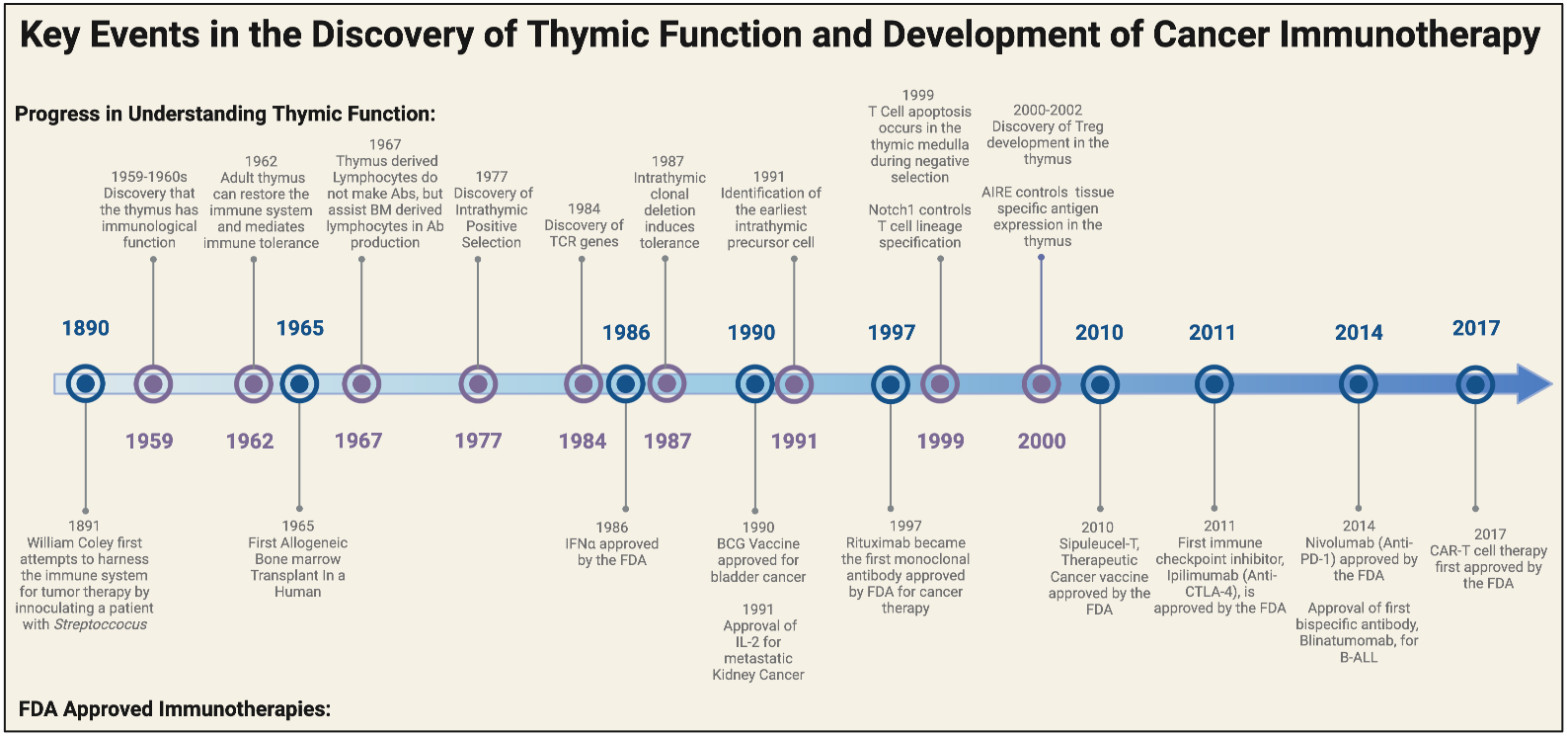
Historical timeline of major discoveries on the thymus and the development of FDA approved cancer immunotherapies. Major breakthroughs in understanding the functional role of the thymus began in the late 1950s to early 1960s. These landmark discoveries paved the way for breakthroughs in T cell biology and eventually ushered in a new era for cancer immunotherapy. Elements of this figure were generated using Biorender.com.

For centuries, the thymus was misconceived to be a vestigial organ or at most a ‘graveyard’ for old and dying lymphocytes. The observation that adult mice who underwent thymectomy had no defects in immune response and that the thymus shrank after infection and in adulthood initially suggested to many scientists that its function was inconsequential to the immune system. However an important paradigm shift occurred in the early 1960s, when Jacques Miller discovered that the removal of the thymus during neonatal development could cause marked lymphocyte deficiency (17). This was the first demonstration that the thymus played an essential role in immunological function and paved the way for fundamental discoveries in our understanding of T cell development, potentially transforming the way we practice modern medicine (**Figure 1**).

**Figure 2:**
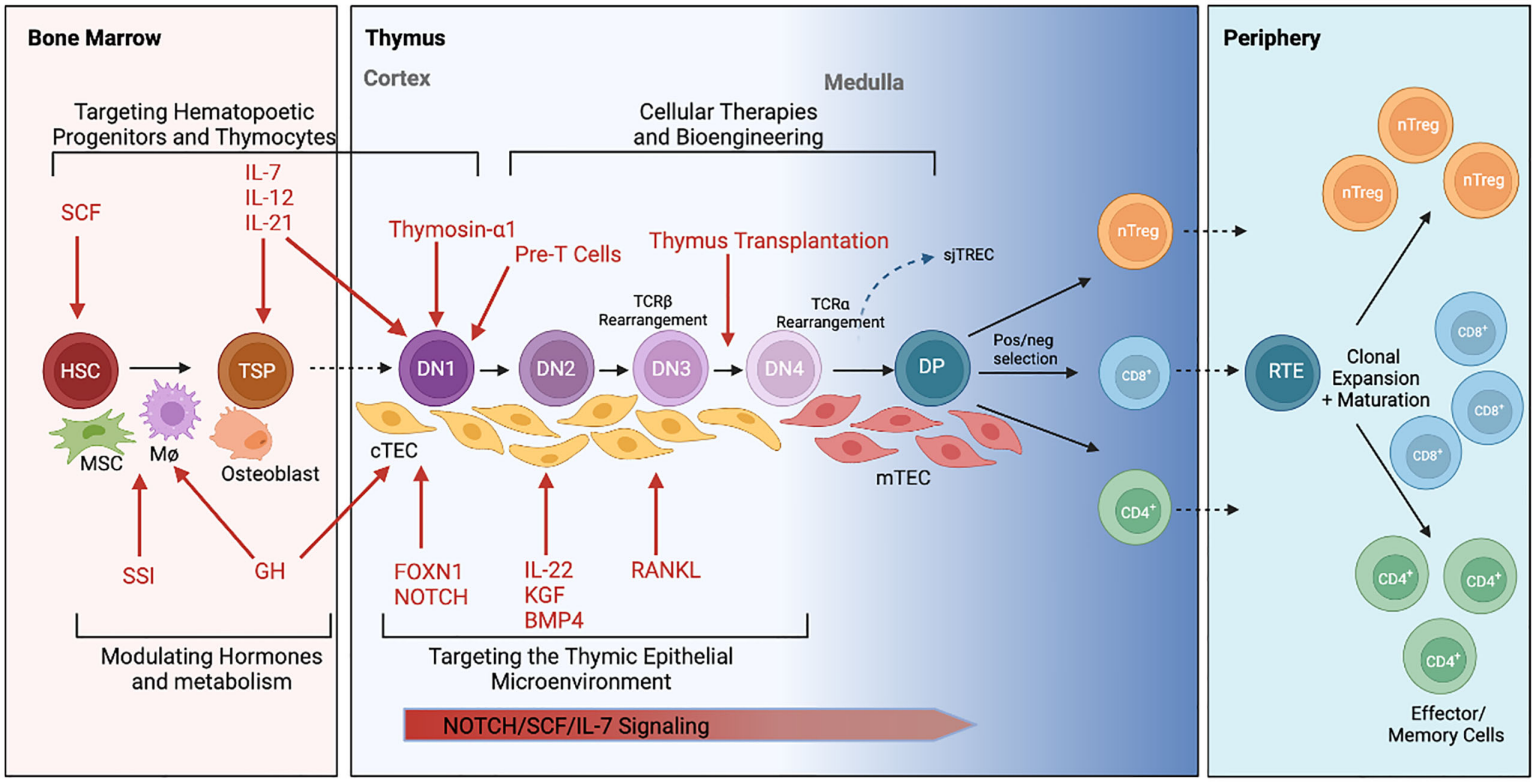
Schematic illustration of intrathymic T cell development and current approaches to thymus rejuvenation. T cell development begins with migration of hematopoietic stem cells (HSC) from the bone marrow to the thymus. These thymic seeding progenitors (TSPs) undergo lineage commitment as they transform from double negative (DN) to double positive (DP) to single positive T cells with the assistance from critical signals within the thymic epithelial microenvironment. T cells are eventually exported out of the thymus as CD4+, CD8+ or regulatory T cells (Tregs) where they function within the periphery. Various therapeutic approaches (in red) to rejuvenating thymic function are currently under investigation. This includes approaches to 1) target hematopoietic progenitors and thymocytes, 2) target the thymic epithelial microenvironment, 3) modulate hormones and metabolism, and 4) develop cellular therapies to replace thymic tissue. Elements of this figure were generated using Biorender.com.

Since the discoveries made by Miller, it is now accepted that the thymus is the primary site of T cell development and is essential for maintaining homeostatic cellular immunity, central tolerance, and tumor immunity (9). The thymus coordinates the development of cell-mediated antitumor immunity through the generation of a diverse T lymphocyte repertoire capable of recognizing tumor neo-antigens (18–20). The acquisition of a tumor reactive T-cell population is orchestrated *via* cross talk between the bone marrow microenvironment and the stromal thymic microenvironment, which is formed by a meshwork of medullary and cortical thymic epithelial cells (mTECs/cTECs), macrophages, dendritic cells, fibroblasts and matrix molecules (**Figure 2**) (21).

T cell lineage identity is determined by a complex regulatory network of genetic interactions that are initially triggered by environmental signals and metabolic crosstalk (22, 23). Hematopoietic precursors from the bone marrow migrating to the thymus, termed thymocytes, receive a series of complex but critical signals (e.g. Notch ligands, Interleukin (IL)-7, and stem cell factor) from TECs to promote the survival and lineage commitment to CD4 or CD8 single positive T cells (**Figure 2**) (24, 25). The generation of unique T cell receptors (TCRs) able to recognize diverse antigenic peptides is a crucial step for T-cell development in the thymus and involves the somatic rearrangement of three complementary-determining regions (CDRs) of the TCR (26). During each step of T-cell receptor rearrangement, DNA fragments between rearranged gene segments are deleted as circular signal joint T cell receptor excision circles (sjTRECs), which are frequently evaluated using RT-PCR to quantify thymic activity (27).

Finally, the survival and differentiation of T cells is determined by positive and negative selection induced by interactions between TCR and major histocompatibility complex (MHC)/self-antigens. Strong TCR signals lead to clonal deletion of potentially dangerous, highly self-reactive CD4+ T cells, whereas weak signals lead to generation of conventional T cells (28). Prior to exiting the thymus, a proportion of CD4+ T cells with intermediate TCR signals differentiate into forkhead box P3 (FOXP3) expressing regulatory T cells (Tregs) (29, 30).

## 3 Age related thymic involution and AML

Age-associated alterations to the immune system result in progressive immunosenescence and is believed to potentially contribute to the increasing incidence of cancer in older patients (31, 32). Similarly, patients with advanced age constitute a large subset of patients with AML and are more often found to have unfavorable cytogenetics, multidrug resistance, and poor prognosis. This may suggest that there are differences in AML biology that occur with aging (33–35).

The physiological involution of the thymus after the first year of life is perhaps the most pronounced change to the aging immune system in humans (36). This is characterized by the profound perturbation of the thymic stromal microenvironment, including the loss of thymic epithelial cells and progressive replacement of healthy tissue with adipose tissue (37, 38). While the atrophied thymus is still capable of producing new T cells, age-related involution is well known to severely compromise naïve T cell output and perturb signal strength between thymocyte TCR and mTEC MHC-II, resulting in impaired thymic negative selection and enhanced thymic Treg generation (20). Thymic involution has additionally been shown to result in the expansion of oligoclonal peripheral memory T cells, thereby restricting TCR repertoire diversity and skewing type 1/type 2 cytokine production profiles (39, 40).

Reduced T cell-mediated tumor immune surveillance is a likely consequence of these age-related changes and may at least partially explain the clinical and biological differences of AML in older patients. A recent survey of pre-treated AML BM using multiplex immunohistochemical analysis demonstrated 2 major immunologic AML clusters differing significantly in age (median 54.8 vs 64.6 years). AML patients in the older cluster were observed to have higher TCR clonality as well as higher proportions of OX40+ and memory CD45RO+ T cells. Importantly, the higher TCR clonality in the older cluster correlated with higher age and worse prognosis (41). Thus, it’s possible that age-related thymic involution is one of the causative mechanisms leading to the decline in T cell-mediated tumor immune surveillance in the elderly who are developing AML (20, 36, 42–44).

Two primary mechanisms have been proposed to explain the observed aged-related decrease in thymopoiesis (45). Firstly, it has been proposed that diminished HSC output and increased rate of apoptosis with age reduces the number of thymus seeding progenitors (TSPs) entering the thymus from the BM. Aging TSPs have been shown to have reduced expansion and differentiation potential through increased *Ink4a* expression and diminished CD3 expression in DP and SP thymocytes (46–49). Secondly, aged induced defects in the thymic stromal niches also results in defects in thymopoiesis (50, 51). Alterations in thymic architecture are observed as early as puberty, when thymic atrophy is the greatest (52, 53). With increased age, the thymus undergoes notable reduction in TECs, blurred demarcation of thymic cortical and medullary compartments, downregulation of various TEC markers, as well as increased fibrosis and adipose deposition (54–56). Taken together, we hypothesize that these age-related physiological changes including impairments in both recruitment of TSPs and maintenance of thymic architecture might increase the risk of the development of AML in elderly patients.

## 4 Effect of thymic function on the tumor microenvironment

The dynamic interactions between AML cells and the tumor microenvironment (TME) create a specialized niche to support leukemogenesis and immune evasion (57). Compared to solid tumors, the leukemic TME inherently involves the bone marrow, which serves as a sanctuary for leukemic stem cells, extracellular matrix, stromal cells, immune cells and the soluble factors they secrete (58). Given its role in maintaining T cell repertoire and central tolerance, the thymus is another primary lymphoid organ that should be considered for its role in influencing the AML TME, especially with respect to shaping the T cell milieu. Age-related thymic involution has not been associated with any clinical consequences in healthy individuals, perhaps because residual thymic activity can still persist beyond the seventh decade of life and influence T cell responses, especially after immunological injury (10, 59).

### 4.1 Thymus influences regulatory T cell populations

Recently, CD4+ CD25+ FOXP3+ Tregs have gained attention for their immunosuppressive capabilities in the AML tumor microenvironment. Accumulating evidence has shown that proportions of Tregs are increased in the peripheral blood and bone marrow of AML patients and are associated with poorer prognosis (41, 60–63). In addition, current research suggests that Tregs promote leukemia cell survival by suppressing both expansion and effector functions of cytotoxic T lymphocytes, as well as directly promoting the stemness and survival of AML blasts through the IL10/PI3K/AKT signaling pathway (64–66).

Tregs are a heterogenous population and can be both naturally derived in the thymus (nTregs), which is believed to make up approximately 80% of Treg repertoire, or induced by conversion of conventional T cells existing in the periphery (iTregs). Whether Tregs within the tumor microenvironment are natural vs induced remains a subject of debate, as they are difficult to distinguish *in vivo* (67). However, several biomarkers such as Helios, NRP1, and FOXP3 methylation status have been reliably used to identify Tregs of thymic origin (68–70). In this regard, a previous study showed that expanded Tregs from AML patients treated with histamine dihydrochloride (HDC) and low dose IL-2 for relapse prevention predominantly resembled nTregs, based on Helios expression and TSDR methylation status in the FOXP3 gene locus (71). These findings corroborate a previous study showing that a majority of tumor-infiltrating Tregs are thymically derived and reactive to Aire-dependent self-antigens associated with the organ of cancer origin (72). Together, these studies have important clinical implications because thymic involution from aging or chemotherapy has been shown to enhance the generation of nTregs (73).

### 4.2 Thymus’ role in clonotype TCR diversity

As we have previously discussed, thymus involution has been known to result in limited TCR diversity. Several groups have similarly reported restricted TCR repertoire diversity and skewed oligoclonal αβ^+^ and γδ^+^ T- cell pools in AML (74–77). Notably, recent studies have shown restricted expression and oligoclonal expansion of certain Vβ subfamily T cells, such as PD-1^+^ Vβ T cells in patients with AML and CML (76, 77). The oligoclonal expansion of certain TCR γδ T cells from patients with AML have likewise been reported (78). Although γδ T cells are less studied and constitute a smaller percentage of the human T cell population, there is growing evidence to support its role in antitumor immunity (79). In fact, Vγ9Vδ2 T cells have been shown to specifically recognize and kill AML blasts in a TCR dependent manner (80). A study comparing TCR repertoires between pediatric and adult AML showed that the fraction of γδ T cells increases with age, which is the inverse of what is seen in healthy patients (74). Taken together, it is likely that certain leukemic-specific T cell clones in the TME may be preferentially expanded by AML-associated antigens in the periphery and/or are being restricted during development in the thymus. In support of the latter, a recent study found that effector functions of γδ T cell subsets within the TME was dictated by metabolic programing during early thymic development (81).

### 4.3 Thymic involution promotes inflammaging and the immunosuppressive TME

Age-related thymic atrophy has been correlated to the development of systemic inflammation with advanced age, termed inflammaging (45). Recent studies also suggest that mTEC dysfunction with age reduces the capacity to eliminate self-reactive thymocytes through impaired negative selection (82, 83). This process has been proposed to potentiate the increasing self-reactivity and auto-inflammation observed with aging (45). It is known that chronic inflammation can aid in tumor progression, metastasis and drug resistance in tumor cells (84), and pro-inflammatory mediators also promote the disease progression and relapse of AML (16, 85). Accordingly, it is thus possible that the disruption in T cell homeostasis derived from thymic atrophy might play a role in AML pathogenesis.

### 4.4 Thymus’ role in sustained CD8 T cell immunity during chronic antigen exposure

Previous studies have shown that CD8+ T cells are functionally impaired in AML including expressing aberrant phenotypes, having increased expression of exhaustion/senescence molecules (CD57, TIGIT, TIM-3 and PD-1), and forming defective immune synapses with blasts (11, 12, 86–90). While thymic function gradually decreases with age, continued thymopoiesis in residual thymus tissue has been shown to be essential for the generation of new CD8+ T cells to replenish attrition from exhausted T cells during chronic antigen exposure. This has been demonstrated in patients with chronic polyomavirus infection who depend on the reactivation of thymic function and *de novo* T cell generation to sustain antigen-specific CD8+ T cell immunity (91).

## 5 AML interferes with intrathymic T-cell development

AML has been shown to promote thymic atrophy, suggesting that AML may somehow damage the thymus and its production of functional T lymphocytes (92, 93). A dramatic reduction of sjTREC was found in peripheral T cells of AML patients when compared to age-matched healthy controls, raising the possibility that immunodeficiency in AML could be derived from the diminished thymic output of T cells (92). Furthermore, AML patients with high sjTREC counts have better disease prognosis, and the sjTREC counts were found to recover to normal values in those patients achieving complete remission (92, 94). Consistent with these clinical findings, another study observed that immune-competent mice challenged with AML blasts also developed premature thymic atrophy, which was characterized by reduced numbers of thymocyte subsets, most notably the DP population (93). Thymic atrophy resulted in significant loss of peripheral CD4+ and CD8+ T-cells with an increased frequency of CD4+Foxp3+ regulatory and activated/memory T cell subsets. The expression of monocyte chemoattractant protein 1 (MCP-1/CCL2) was found to be associated with thymic atrophy and the neutralization of CCL2 *via* an anti-CCL2 pAb enhanced antileukemic T-cell response and increased the survival of AML mice (93).

## 6 Can AML originate in the thymus?

AML is believed to originate from transformed myeloid restricted progenitors residing within the bone marrow (95). However, the existence of acute leukemia expressing both myeloid and lymphoid lineage-specific markers, known as mixed-lineage acute leukemia (MPAL), suggests that at least some blast populations may originate from leukemia stem cells possessing multilineage potential (96). By the same token, thymus-seeding progenitors (TSPs) have largely been regarded to have a restricted lineage towards T-lymphocytes. Yet several recent studies have shown that double negative (DN) TSPs may retain the potential to differentiate to alternative hematopoietic fates, including NK, B-cell, and myeloid lineages (97–101). Transformed DN2 murine T-cell progenitors overexpressing oncogenes *Myc*/*Bcl2* have been shown to have high intrinsic potential to transdifferentiate into myeloid and biphenotypic leukemia. Remarkably, the resulting murine DN-2 derived leukemia was found to be genetically similar to a human AML cohort (102). Moreover, murine DN2 with a known human NUP98-HOXD13 (NHD13) fusion transcript have a predisposition to transform into a highly aggressive AML with similar gene expression profiles of several human AML subsets, including those with NPM1 mutations, MLL fusions and NUP98 fusions (103, 104). While rare, several case reports have described AML with involvement of the thymus/mediastinum without evidence of BM infiltration (105–108). Together, these studies pose the possibility that the thymus can be involved in the leukemogenesis of AML, and some subsets of AML might originate from thymic precursors.

## 7 Implication of thymic function on current therapies for AML

### 7.1 Cytoreductive therapies impair *de novo* T Cell recovery

The thymus is exquisitely sensitive to cytoreductive therapies that are traditionally used as the standard of care in AML, such as chemotherapies or radiation therapies. While all hematopoietic cell types are affected following chemotherapy, T cells appear to be most profoundly impacted, likely in part due to depletion of thymocytes and TECS within the thymic stromal compartment (109, 110). The recovery of the depleted T cell pool after cytoreductive therapy may take many years or be permanent (111, 112). Age-related thymic involution has also been linked to impaired immune reconstitution following chemotherapy, which may partially explain treatment failure in the elderly (113–115). Considering the impact of cytoreductive therapies on thymic function and *de novo* T cell output, adoptive T-cell based immunotherapies utilizing bioengineered allogenic or autologous T cells, such as CAR T cell therapy or tumor-infiltrating lymphocytes (TILs) may be a promising therapeutic approach, either as a supplement to first-line treatments or as a treatment for refractory cases (14, 116).

### 7.2 Chemotherapy- induced thymic atrophy creates a chemoprotective tumor reservoir

Relapsed and refractory disease presents a significant challenge and is primarily responsible for the poor prognosis in AML. The emergence of remnant populations of leukemic cells after exposure to chemotherapy, termed measurable residual disease (MRD), implies that AML cells may develop drug-resistance or mechanisms to evade exposure to therapy. Several premetastatic reservoirs, such as the bone marrow and the perivascular space of blood vessels, have been identified as potential reservoirs that permit primary tumor cells to hide following adjuvant chemotherapy (117–119). When damaged by chemotherapy, the atrophied and inflamed thymus has likewise been found to create a chemoprotective microenvironment for both solid and hematologic tumor cells (120–122). Notably, the release of the proinflammatory cytokines TIMP1 and IL-6 from TECs in response to cytotoxic chemotherapy has been shown to induce TEC senescence and promote lymphoma cell survival (121). Exposure to chemotherapy also induces thymic residing tumor cells to acquire an antiapoptotic chemo-resistant phenotype within the inflammatory thymic microenvironment (120). Finally, the presence of tumor cells within the thymus may also interfere with *de novo* T cell differentiation and lead to tumor-specific immune tolerance. This hypothesis is supported by several studies that have shown that infectious pathogens that invade the thymus lead to microbe-specific T cell tolerance (123–127). Together, these findings suggest that the atrophied thymic microenvironment may be chemoprotective to AML blasts, harbor MRD following adjuvant therapy, and result in tumor-specific T cell tolerance that leads to an eventual tumor relapse.

### 7.3 FLT3 ligand and CXCR4/CXCL12 regulates thymic precursors

*FMS-like tyrosine kinase 3* (FLT3) is widely expressed by immature hematopoietic progenitors and over-expressed in a majority of AML blasts, often in the presence of activating mutations by tandem duplications (FLT3-ITD) and/or point mutations involving the tyrosine kinase domain (TKD) (128). The presence of *FLT3-ITD* is widely accepted to portend a poor prognosis in AML due to chemoresistance and the high rate of relapse. Recent data has suggested that FLT3 and its respective ligand (FLT3L) may be an important point of regulation for thymic function. FLT3L knockout mice were notably observed to have reduced immature thymocytes and Lin^-^SCA1^+^KIT^+^ (LSK) lymphoid-primed multipotent marrow progenitors (129, 130). More importantly, administration of FLT3 ligand was found to enhance the export and survival of LSK cells, early thymic progenitors after transplantation, and androgens *via* the downregulation of CXCR4 (131). Potentially, these findings have important clinical implications because the use of FLT3 inhibitors after HSCT may impair thymic recovery in AML.

### 7.4 Allogeneic hematopoietic stem cell transplantation

Allo-HSCT is currently the only curative treatment option for AML; though, its success both in regards to its ability to fight opportunistic pathogens and eliminate leukemia depends on optimal recovery of adaptive immunity after cytoreductive conditioning (132). Thymic-independent proliferation of donor-derived T-cells can initially restore peripheral T-cell numbers within the first year following Allo-HSCT. However, because these T-cells are derived from a limited number of donor precursors, their TCR repertoire is of limited diversity (133–136). This has important clinical implications, as lower TCR diversity has been associated with increased risk for disease relapse (137). Therefore, complete T-cell reconstitution with a diverse TCR repertoire may depend on the recovery of *de novo* T-cell production in the recipient thymus (132, 136, 138). A recent study has importantly demonstrated that some donor-derived CD8 T cells specific for hematopoietic cell-restricted antigen can escape deletion in the thymus and contribute to GVL effects in the periphery (139). Considering that thymic function is also highly sensitive to pre-transplant conditioning regimens, the above findings provide a rationale to developing strategies to boost thymic recovery to improve the outcome of Allo-HSCT (140).

### 7.5 Graft- versus- host disease

GVHD is a common but significant complication of Allo-HSCT and has additionally been shown to interfere with thymic-dependent T cell development by reducing TSP as well as altering thymic cellularity and architecture (138). This is in part mediated by a glucocorticoid-independent mechanism of DP thymocyte apoptosis, as well as the destruction of mTECs *via* donor alloreactive T cells by expression IFN-gamma, and the cognate proteins FasL and TNF-related apoptosis-inducing ligand (TRIAL) (138, 141–147). The capacity to affect thymic output after HSCT has been demonstrated by the observation that acute GVHD (aGvHD) is associated with a significant reduction in sjTREC counts (148–150). In chronic GvHD (cGvHD), research has demonstrated that sjTREC levels remain reduced even after recovery and long-term follow-up. However, this effect was transient in young patients <25 years old, highlighting the age-dependent regenerative capacity of the thymus (150).

### 7.6 Therapeutic cancer vaccines

Therapeutic cancer vaccination to induce remission or prevent relapse has been extensively explored in preclinical and clinical trials over the last decade (151). However, the efficacy of tumor vaccination strategies may be compromised by several tumor extrinsic factors secondary to thymic atrophy, including Treg driven immune suppression, T cell exhaustion, and reduced TCR repertoire. Though the role of thymic atrophy on anti-tumor vaccines has yet to be studied directly, age-related thymic involution has been cited as one cause for the diminished response to traditional vaccination. This has been suggested based on the observation that only an estimated 30-40% of elderly patients are able to mount an adequate immune response to the influenza vaccine (152).

### 7.7 T-cell directed Immunotherapy

The success of immunotherapy in AML is contingent on the presence of functional T cells capable of recognizing tumor specific antigens. Additionally, the degree of neoantigen heterogeneity, tumor mutational burden, and TCR repertoire diversity has been demonstrated to increase the likelihood of tumor antigen recognition and improve overall survival (153–155). Considering the role of the thymus in maintaining homeostatic T-cell immunity, it is likely that a decline in its function could compromise the efficacy of T-cell directed immunotherapies in AML.

The use of immune checkpoint inhibitors (ICIs) in AML, such as anti-CTLA-4 antibody ipilimumab, and the anti-PD-1 antibody nivolumab have demonstrated limited response compared to its impressive efficacy in solid tumors (156–158). Several strategies have been employed to predict response to ICI in solid tumors, including TCR repertoire profiling by high-throughput sequencing before and after ICI treatment (159). Previous studies in melanoma, gastrointestinal cancers, and relapsed/refractory classical Hodgkin lymphoma have overall shown that a broader TCR profile is associated with superior outcomes in patients receiving ICIs (160–164). Consistently, a recent paired analysis of single cell RNA and TCR repertoire profiling in relapsed/refractory AML demonstrated that TCR repertoires primarily from CD8+ cells expand in patients who responded to PD-1 treatment, but contract in those who were treatment resistant (165). Perhaps assessment of TCR repertoire and even the functional status of the thymus can be used to risk stratify and monitor patients receiving ICI therapy and thereby determine optimal personalized treatment strategies for AML patients.

While the application of genetically engineered CAR-T-cell therapies have been successful for lymphoid malignancies, its use for the treatment of heterogeneous AML faces several unmet challenges (166). This includes the lack of AML-specific cell-surface antigen that minimizes off target toxicity and the suppression of T cell activity and proliferation by AML blasts (167). As previously discussed, the increased Treg frequency following thymic atrophy creates a hostile tumor microenvironment for CAR T cells who are already susceptible to exhaustion *via* chronic tumor antigen exposure. There is some research to suggest that mature peripheral T cells can re-enter the thymus, localize to the medulla, and alter the stromal microenvironment (168, 169), which was attributed to the onset of thymic involution (170). Remarkably, adoptively transferred syngeneic antigen-specific T cells in lymphopenic mice has been shown to enter the thymus and eliminate thymic dendritic cells (DCs) and mTECs presenting cognate antigen (171). These findings have important clinical implications, as CAR T cells may eradicate tumor antigen expressing APCs that mediate negative selection, thereby enhancing the patient’s endogenous antitumor repertoire. However, given that most AML antigens may also be expressed by normal HSPCs and healthy tissue, caution must be used, as this phenomenon may also augment the risk for autoreactivity *via* on-target, off-tumor toxicity (116).

## 8 Strategies to rejuvenate and boost thymic function

As discussed above, the thymus is important for the development of T cell mediated tumor immunity in AML, and boosting its function may be a promising route for improving the efficacy of future therapies. In this regard, several strategies are currently being investigated to rejuvenate thymic function, including approaches to target the TEC microenvironment, thymic/hematopoetic progenitors, thymic growth/metabolism, as well as approaches to generate or transplant new thymus tissue. While few have been successfully translated for clinical use, their use in combination with cancer immunotherapies may be a potential avenue for future research (113, 172). Here, we review promising approaches to improving thymic function within the AML microenvironment landscape (**Table 1**).

**Table 1:** Current approaches to Thymic/T cell regeneration in AML and other hematologic malignancies.

Therapeutic target	Clinical trials	References
*Targeting the Thymic Epithelial Microenvironment*		
IL-22	NCT02406651	(173–175)
KGF	NCT01233921, NCT03042585, NT02356159, NCT00593554, NCT01712945, NCT00031148, NCT00056875, NCT00004132, NCT00070616, NCT00004061, NCT00189488, NCT00041665, NCT00109031, NCT01746849 NCT00482846, NCT00376935	(176–188)
RANKL	Not currently in clinical trial	(189–191)
FOXN1	Not currently in clinical trial	(192, 193)
GH and Ghrelin	NCT00071240, NCT00119769, NCT00287677, NCT00050921, NCT04375657	(188, 194–199)
BMP4	Not currently in clinical trial	(200–203)
Notch Ligand		(204, 205)
*Targeting Thymocytes and Hematopoietic Progenitors*		
IL-7	NCT00477321, NCT01241643, NCT01190111, NCT00684008, NCT00839436, NCT02293161, NCT03941769	(206–216)
IL-12	NCT02483312, NCT00003210, NCT00003149, NCT00003107, NCT00004260, NCT00003575, NCT00003330	(217, 218)
IL-21	NCT04220684, NCT00347971, NCT02809092, NCT01787474	(205, 219–221)
GH and Ghrelin	As above	
Thymosin-α1	NCT00580450	(187, 222)
SCF	Not currently in clinical trial	(184–186)
*Modulating Hormones and metabolism*		
SSI	NCT01746849, NCT01338987	(48, 109, 223–235)
Antioxidant	Not currently in clinical trial	(236)
*Cellular Therapies and Bioengineering*		
Artificial thymus scaffold	Not currently in clinical trial	(237–240)
Thymic epithelial progenitors	Not currently in clinical trial	(241–246)
Precursor T cells	Not currently in clinical trial	(204, 247–256)

Clinical trials obtained through advanced search of clinicaltrials.gov.

IL, interleukin; KGF, keratinocyte growth factor; RANKL, receptor activator of nuclear factor-kB ligand; FOXN1, forkhead box n1; GH, growth hormone, BMP4, bone morphogenic protein 4; SCF, stem cell factor; SSI, sex steroid inhibition.

### 8.1 FOXN1

As previously discussed, the thymic stromal compartment is crucial for intrathymic T cell development. The expression of key transcription factor, Forkhead box N1 (FOXN1) is important for TEC differentiation, thymic organogenesis during embryonic development, antigen processing, thymocyte selection, and has been implicated in regulating age-related thymic involution (257–260). While downregulation of *foxn1* gene with age results in functional decline in the TEC compartment, its overexpression has been shown to delay thymic degeneration (257, 261). A study by Bredenkamp and colleagues showed that increased FOXN1 expression is sufficient to drive regeneration of the aged thymus both in regards to its architecture, gene expression and functionality (192). More recently, Oh et al. demonstrated embryonic fibroblasts reprogrammed to overexpress FOXN1 could be engrafted to rejuvenate thymic function in mice (193). While there are currently no ongoing clinical trials, developing therapies which target FOXN1 expression may provide one strategy for regenerating an aged thymus further adversely affected by chemotherapeutic regimens used to treat AML.

### 8.2 Interleukin-7

Interleukin-7 has come into focus as an important non-redundant regulator of lymphopoiesis and mature T cell homeostasis through both thymic dependent and independent mechanisms (10, 262). In the thymus, IL-7 promotes the survival of DN thymocytes, TCR rearrangement, and lineage differentiation during positive selection (263). Furthermore, IL-7 expression appears to decline with age, correlate with thymic atrophy, and importantly appears to be down-regulated in the peripheral blood of AML patients (264–266). The use of exogenous IL-7 for immune reconstitution has been extensively investigated in several preclinical and clinical studies (262). Administration of IL-7 in mice after T-cell depleted allo-HSCT was found to significantly expand donor-derived thymocytes and peripheral T cells, but remarkably had no effect on alloreactive T cells and the development of graft-versus-host disease (206, 207). Several clinical trials have also evaluated the use of glycosylated recombinant human IL-7 (CYT107) in HIV-1 infected patients and found that IL-7 treatment was not only safe and well tolerated, but could also enhance thymopoeisis as demonstrated by an increased number of recent thymic emigrants (RTEs), increased TREC ratio, and increased TCR repertoire diversity (208–210). Notably, these findings were addressed in a phase 1 clinical trial in which CYT107 was used in recipients of T-cell depleted allo-HSCTs; unfortunately, changes in RTE or TREC levels were only appreciated in a small subset of patients (211).

### 8.3 Keratinocyte growth factor

Keratinocyte growth factor (KGF) is a potent mitogen expressed by thymic mesenchymal and stromal cells that mediates TEC proliferation and survival through activation of the PI3k-AKT-nuclear factor-kB and p53 pathways (176, 177). Several studies have demonstrated that treatment with exogenous KGR could avert GVHD-related injury, enhance thymopoiesis in HSCT recipients and protect TECs during irradiation-induced injury (176, 267, 268). Furthermore, the use of KGF could reverse age-related thymic involution and restore thymopoiesis in aged mice for up to 2 months after treatment (269). The use of recombinant KGF (palifermin) has since been approved by the US Food and Drug administration for the treatment of oral mucositis in patients receiving intensive chemotherapy (270). Currently, there are multiple clinical trials underway to further investigate the use of palifermin for T cell reconstitution (NCT01233921, NCT03042585, NT02356159 and NCT00593554).

### 8.4 Sex steroid inhibition

The observation that thymic function rapidly declines after puberty and that castration rejuvenates thymic function has suggested that sex hormones, particularly androgens, play a role in thymic involution (48, 271–273). Consistent with these findings, several studies have demonstrated that sex steroid inhibition (SSI) promotes thymopoeisis by increasing TEC expression of CCL25, Dll4, and Notch signaling pathways (223, 224). The regenerative impact of SSI has further been found to enhance immune function in immunocompromised patients or those undergoing auto-HSCT (274, 275). To date, several drugs have been developed to transiently inhibit sex steroids, such as luteinizing hormone-releasing hormone (LHRH) antagonists, for use in precocious puberty, prostate cancer, breast cancer, and endometriosis. More recently, two clinical trials (NCT01746849 and NCT01338987) are underway to evaluate the effects of SSI on immune reconstitution following allo-HSCT. A pilot study using LHRH agonist (goserelin) administration 3 weeks prior to HSCT has already demonstrated significant increases in naive CD4+ T cells, TRECS, and recovery of TCR repertoire diversity (225).

### 8.5 Growth hormone

The expression of growth hormone (GH) progressively declines after the third decade of life and has been linked to both hematopoietic and thymic function (194, 276, 277). Given its immunomodulatory effect in humans, several preclinical and clinical trials have investigated the use of exogenous GH to boost immune function and demonstrated that it can reverse thymic atrophy and improve TCR diversity (194–196). Notably, two prospective randomized trials reported that daily recombinant GH injections for 6-10 months could enhance thymic output and TREC levels in HIV-1 infected patients (195, 278). Another trial also remarkably showed that GH treatment reduced PD-1^+^ CD8 T cells, suggesting that this could also be used to reverse CD8 T cell exhaustion (197). While GH therapy has been approved for use in pediatric patients who develop post-radiation growth disorders after HSCT, GH therapy has yet to be implemented for immune reconstitution (279, 280). Before it may be used for thymic reconstitution, several concerns must be addressed. Firstly, it appears the effect on thymic output appear to only be transient, as discontinuation of GH results in recurrence of thymic atrophy (196). Secondly, GH is associated with many undesirable side effects, including increased risk for cardiovascular disease, stroke, and diabetes. Importantly, GH and growth hormone-releasing hormone (GHRH) have recently been implicated in AML proliferation (281, 282). These side effects reduce the enthusiasm for the use of GH or GHRH in treating AML.

### 8.6 Notch ligands

The expression of Notch ligands Delta-like-1 and 4 (DLL4 and DLL1) by cTECs is essential for supporting T-lineage commitment (283). In the absence of notch signaling, thymopoiesis is arrested early in T cell development during double negative thymocyte differentiation (284). Conversely, its constitutive activation in HSPC simultaneously results in inhibition of B cell development and promotion of T cell development toward double positive status in the thymus (285). Furthermore, a recent study by Tikhonova et al. investigating the transcriptional changes of the BM microenvironment in response to chemotherapy demonstrated that DLL4 and DLL1 is notably downregulated by the vascular endothelium in response to stress (286).

The potential for Notch signaling for T cell reconstitution has been explored in several studies. For instance, the treatment of lin^-^ Sca-1^+^ C-kit^-^ (LSK) hematopoietic progenitors with DLL1 *ex* vivo has been shown to accelerate thymus engraftment and T cell reconstitution after HSCT (204). More recently, it has been shown that activation of a BM-specific Notch/IL-21 signaling axis could lead to *ex vivo* expansion of T cell progenitors (205). It is important to note that Notch has also been cited as a tumor suppressor in myeloid malignancies and that reduced Notch signaling may play a role in skewing HSPC to premature myeloid lineages that could progress to leukemic myeloid production (287–289). Together, these findings provide a rationale to develop strategies to improve for T cell regeneration that utilize Notch and its downstream signaling pathways for use in treatment of AML.

### 8.7 Bone morphogenic protein-4

Bone morphogenic protein-4 (BMP4) is a member of the TGF-β superfamily, known for its role in regulating embryonic development (290). However, new research has highlighted its function in thymic tissue regeneration after injury (200, 201). A recent study by Wertheimer et al. showed that TECs increased expression of BMP4 after thymic injury, resulting in increased expression of *Foxn1* and its downstream target, Notch ligand *Dll4* (200). More remarkably, they found that inhibition of BMP4 resulted in impaired thymic regeneration, and its exogenous administration could rescue its ability to repair. While not yet in clinical trials, these studies highlight BMP4 as a novel target for improving immunologic recovery.

### 8.8 Cell- based therapies and bioengineering

Adoptive cell-based therapies and *de novo* thymus synthesis have also been investigated for the purpose of enhancing thymic function. Considering that T cell reconstitution is delayed after HSCT due to the limited availability of hematopoetic progenitors, several groups have investigated the potential of supplying donor precursor T cells to boost thymogenesis at the time of transplant (247–249). By expanding hematopoietic precursor cells *ex vivo* using Notch-1 stimulation, researchers have demonstrated significant increases in both thymic cellularity and peripheral T cell reconstitution (250–252). Alternatively, others have attempted to generate new thymus epithelial tissue through the isolation and expansion of thymic epithelial progenitor cells (TEPC) from fetal thymi (241, 242).

Here, we have extensively reviewed the regenerative factors that are currently being studied for repair of the dysregulated immune system, some of which are now being investigated in clinical trials (**Table 1**). We acknowledge the challenge in developing a successful and durable effective immunotherapy for heterogeneous AML in elderly patients, based on the rationale that their declined immune function leads to further disease progression (291). Thus, novel approaches will also be crucial. It has long been recognized from parabiotic mouse studies that there are circulating factors in young mice, which have not yet been identified but which could improve the metabolic and regenerative status of older mice (292). It will then be important to determine if the immune system in the aging mouse also improves with parabiosis. If so, parabiosis could be adapted to studies on the dysregulated immune system in a humanized AML mouse. A serial chronological study of the immune system utilizing the latest genomic techniques, such as single-cell RNA-seq and epigenetic approaches, could disclose key pathways involved in the deterioration of immune regulation with aging and AML. Such mechanistic studies and translational approaches could be applied to both the bone marrow niche and the thymic microenvironment. Searches for important molecular and genetic interactions could be sought as well.

## 9 Concluding remarks

**Figure 3:**
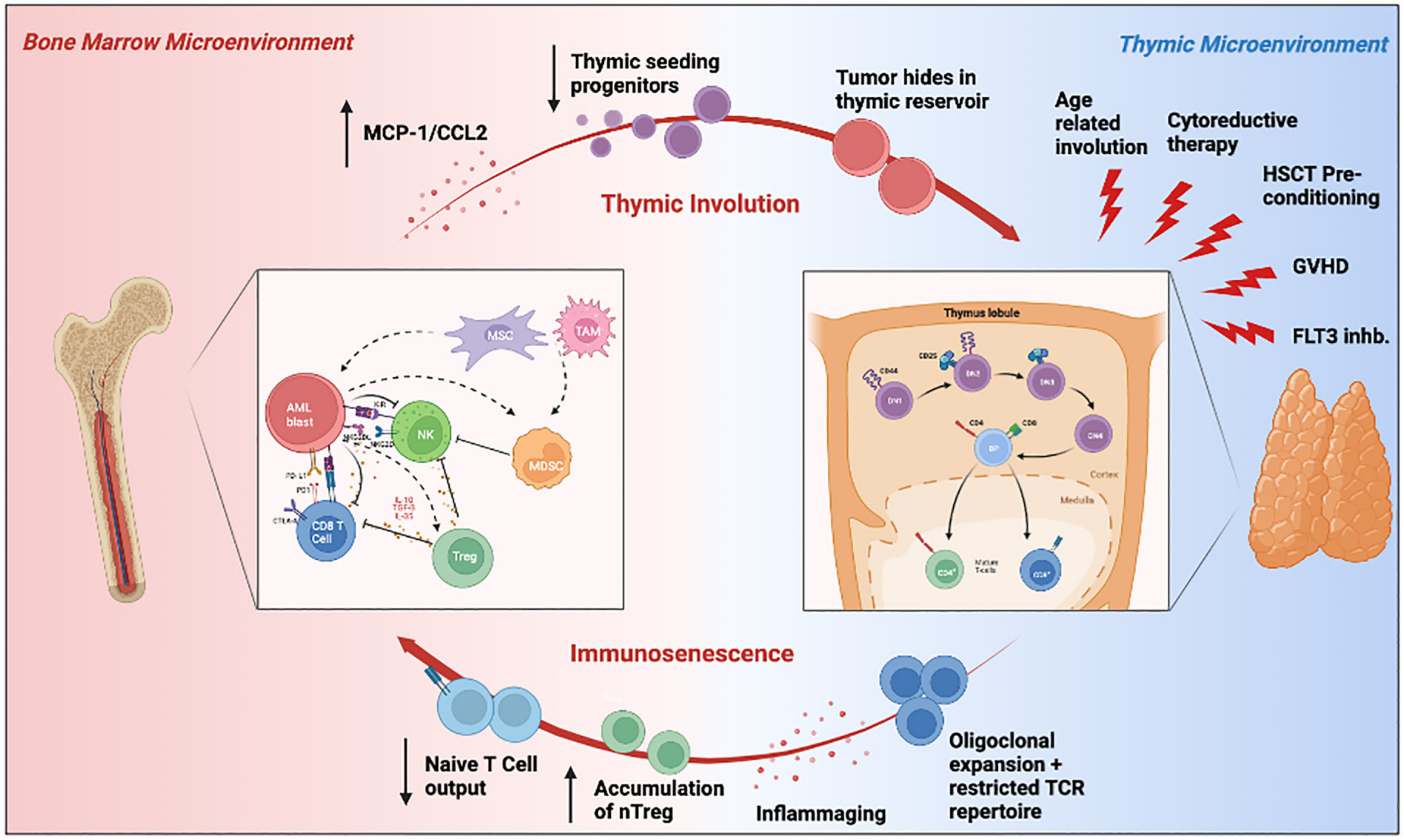
Schematic illustration summarizing the crosstalk between the bone marrow and thymic microenvironment, and its influence on the AML TME. Hematopoietic stem cells derived from the leukemic bone marrow microenvironment can travel to the thymus where they differentiate into T cells. The resulting T cells are then exported to the periphery, where they may then recirculate back into the bone marrow as tumor infiltrating lymphocytes. Environmental insults such as aging, cytoreductive therapy, HSCT pre-conditioning, and FLT3 inhibition may impair thymopoiesis and subsequently promote tumor immune evasion through immunosenscence. The immunosuppressive bone marrow microenvironment can also independently impair thymic function through MCP-1/CCl2 expression and reduction in thymic seeding progenitors. Elements of this figure were generated using Biorender.com.

A growing body of research has demonstrated that hematological malignancies are associated with profound dysregulated immune responses in the host, and this may relate to a worse prognosis and a suboptimal response to immunotherapy. Given the unique role of the thymus in shaping the T cell repertoire, it is conceivable that the crosstalk between the bone marrow and thymic microenvironments contributes to AML’s high rate of therapy resistance and disease relapse, and may have wide repercussions on the future of immunotherapy. As we have briefly reviewed, the thymus is a dynamic primary lymphoid organ impacted by many factors in AML patients, including age, availability of HSC progenitors, treatment with chemotherapeutics or HSCT, and the immunosuppressive tumor microenvironment (**Figure 3**). At the same time, the thymus influences the composition of the cellular milieu in TME. It is thus reasonable to postulate that perturbations to either the BM or the thymic microenvironment may affect the other and be at least partly responsible for the observed immunosenescence in patients with AML. The intense interest and scrutiny of T-cell based immunotherapies in hematological malignancies lends impetus to developing novel strategies to overcome T cell immune dysfunction *in vivo* (293). The clinical features of CAR-T cell exhaustion *in vivo* have been investigated by studying cell proliferation, cytotoxicity, and the median persistence of CAR-T cells in peripheral blood, which was found to be within a range of 20 to 617 days (294). Adequate nutrient levels are essential for restoring mitochondrial bioenergetic function, especially for the survival of functional immune cells and recovery from critical illness for cancer patients (295). Whether a rejuvenated thymus can provide such support to host exhausted CAR-T cells and restore their durable anti-leukemic function remains to be uncovered. Also, the success of T cell therapies relies on a diverse T-cell repertoire shaped by the thymus to be effective. Among the complex mechanisms underlying the multifactorial molecular and cellular interactions between immune senescence, cancer immunosurveillance, cancer immune-editing, and cancer initiation and promotion, thymic atrophy represents one piece of the puzzle (32, 296). Therefore, if immunotherapies are to eventually prove successful, one of future pre-clinical and observational studies should focus on improving our understanding of thymic involution with age, its dysfunction in hematological malignancies and novel strategies that can revitalize thymic function and immunosurveillance.

## Author contributions

YX and CH conceived the study. CH prepared the figures, tables and wrote the manuscript. YX, CH, JX, DB, ME, and HC reviewed and edited the manuscript. All authors contributed to the article and approved the submitted version.
